# Utility of the Dependence Scale in dementia: validity, meaningfulness, and health economic considerations

**DOI:** 10.1186/s13195-018-0414-7

**Published:** 2018-08-13

**Authors:** Carolyn W. Zhu, Bote Gosse Bruinsma, Yaakov Stern

**Affiliations:** 1 Brookdale Department of Geriatrics & Palliative Medicine, Icahn School of Medicine at Mount Sinai and JJP VA Medical Center, 130 West Kingsbridge Road, Bronx, NY 10468 USA; 2 James J Peters VA Medical Center, Bronx, NY USA; 3 Axovant Sciences, Inc., New York, NY USA; 4 Cognitive Neuroscience Division of the Department of Neurology, Taub Institute for Research on Alzheimer’s Disease and the Aging Brain, Columbia University Medical Center, New York, NY USA

**Keywords:** Dependency, Disease progression, Severity of illness, Economics

## Abstract

**Background:**

The concept of dependence has been proposed as a unified representation of disease severity to quantify and stage disease progression in a manner more informative to patients, caregivers, and healthcare providers.

**Methods:**

This paper provides a review of the Dependence Scale (DS) as a quantitative measure of Alzheimer’s disease severity, its properties as an outcome measure, a metric of disease progression, and a correlate of medical costs.

**Results:**

The literature supports the notion that the DS is related to, but distinct from, key severity measures, including cognition, function, and behavior, and captures the full spectrum of patient needs. It also presents as a useful measure for assessing disease progression.

**Conclusions:**

Results underscore the importance of the DS as a unique endpoint in Alzheimer’s disease clinical trials, providing important information about the impact of therapeutic interventions. The DS also is a useful measure for economic evaluation of novel interventions aimed at delaying progression.

## Background

The severity of Alzheimer’s disease (AD) and its progression over time are generally captured on scales that measure distinct domains of cognition, function, and behavior. Several issues arise with this approach. While offering clinicians a valuable tool for quantifying mental functions, these scales are often of limited meaningfulness to patients and caregivers, who may be better served with a broad, relatable representation of individualized disease impact. Focusing treatment on one aspect of the disease (e.g., behavior) could result in other aspects being insufficiently addressed, or even adversely impacted [1]. It can be difficult to identify clear milestones that signal clinically relevant disease advancement during AD patients’ gradual decline in ways that are meaningful to patients and caregivers [2].

As a result of these shortcomings, the concept of dependence has been proposed as a unified representation of disease severity to quantify and stage disease progression in a manner more informative to patients, their healthcare providers, and caregivers [3]. Progressive cognitive impairment and behavioral disturbances in AD lead to increasing loss of function and independence. Dependence is thus considered to be related to the degree of impairment in multiple domains, but is distinct and complementary to each.

A number of scales have been developed to measure dependence, of which the Dependence Scale (DS) has been most widely studied [3]. Other instruments developed to measure dependence include the Record of Independent Living (RIL) [4], Independent Living Scales (ILS) [5], the Behavioral Rating Scale for Geriatric Patients (BGP) [6], and the Nursing Care Dependency (NCD) scale [7]. The merits of these scales have been discussed elsewhere [1]. Here, we review the DS as a quantitative measure of disease severity in AD and review its properties as a study outcome measure, a metric of disease progression, and a correlate of medical costs, and thereby its meaningfulness to patients, their caregivers, and other stakeholders.

### Search approach

An initial MEDLINE search of all indexed journals published in English was conducted by using dependence, dependency, or dependence scale in the abstract or title or as MeSH terms and with dementia or Alzheimer in the abstract or title, yielding 117 potentially relevant articles for which full text is available online. A semi-structured review of abstracts was then conducted and excluded 45 articles that included search terms that were unrelated to the current study, e.g., article focusing on alcohol, drug, substance, or environmental dependency, studies in basic science, brain/spinal injury, or conditions other than dementia. We also excluded 23 articles that used activities of daily living (ADLs) exclusively to define dependence, four that focused on Zarit Burden Index, and seven that were published more than 20 years ago (before 1998). Of the 38 remaining articles that described some measure of dependence in dementia or AD, 15 described other measures of dependence and were excluded. Reference lists from the remaining 23 articles that focused on the Dependence Scale were reviewed for potential additional relevant articles.

### The Dependence Scale

The DS is composed of 13 items, which include 11 dichotomous (yes/no) items and two items scored on a three-point scale, indicating frequency of need (never/occasionally/frequently) (Table 1). The total DS score is the sum of all 13 items, which ranges from 0 to 15, where higher scores indicate more dependence (i.e., loss of independence). The 13 items on the DS were based on items found to reflect functional deficiencies important to patients with AD [8]. Individual items on the scale capture patients’ need for reminders and cueing in daily activities and practical activities, as well as the degree to which patients need supervision when awake. As such, several of the assessed needs are not linked to the performance of specific tasks. The items are hierarchical, each item representing the need for increasing levels of assistance. A patient that is dependent on a more severe item should also be dependent on the less severe items on the DS [3]. It should be noted that the DS measures the amount of assistance needed by the AD patient; it does not necessarily indicate how much assistance he or she receives. There may be factors extraneous to AD, such as lack of caregivers, nursing home beds or insurance coverage, socioeconomic and sociocultural factors, as well as country-specific healthcare policies that influence actual amount of care received. The DS can be administered to an individual close to the patient, who is familiar with the patient’s care requirements by an interviewer with minimal training. After scoring the 13 items on the DS, the items can be used to derive a dependence level, ranging from 0 (no dependence) to 5 (complete dependence) (Table 2).

**Table 1:** The Dependence Scale

		Score
A	Does the patient need reminders or advice to manage chores, do shopping, cooking, play games, or handle money?	0/1/2
B	Does the patient need help to remember important things such as appointments, recent events, or names of family or friends?	0/1/2
C	Does the patient need frequent (at least once a month) help finding misplaced objects, keeping appointments, or maintaining health or safety (locking doors, taking medication)?	0/1
D	Does the patient need household chores done for them?	0/1
E	Does the patient need to be watched or kept company when awake?	0/1
F	Does the patient need to be escorted when outside?	0/1
G	Does the patient need to be accompanied when bathing or eating?	0/1
H	Does the patient have to be dressed, washed, and groomed?	0/1
I	Does the patient have to be taken to the toilet regularly to avoid incontinence?	0/1
J	Does the patient have to be fed?	0/1
K	Does the patient need to be turned, moved, or transferred?	0/1
L	Does the patient wear a diaper or a catheter?	0/1
M	Does the patient need to be tube fed?	0/1
	*Dependence Scale Total Score*	/15

Coding for items A/B: No (0), Occasionally (at least once a month) (1) , Frequently (at least once a week) (2)

Coding for items C–M: no (0), yes (1)

**Table 2:** Derivation of dependence level

Level 0	0 to all items
Level 1	Either A, B, or C = 1
Level 2	2 of A, B, or C = 1 OR A or B = 2 OR D = 1
Level 3	E, F, or G = 1
Level 4	H, I, or J = 1
Level 5	K, L, or M = 1

### Psychometric properties of the Dependence Scale

The initial validation for the DS was performed in the Predictors study cohort of patients with mild to moderate AD, in which the DS demonstrated consistency with scales to assess cognitive and functional impairment as well as strong inter-rater reliability (intraclass correlation of 0.90) and acceptable internal consistency (Cronbach’s α of 0.66) [3]. These robust psychometric properties have since been reproduced in several independent studies (Table 3). Content validity of the DS was confirmed in qualitative assessments amongst clinicians, caregivers, and patients in multiple North American and European countries, substantiating the importance of the items on the DS to AD patients and their caregivers [9]. Analyses using cohorts of subjects with mild to moderately severe AD (MMSE > 10) from the Predictors studies, the DADE study, and the ELN-AIP-901 separately confirmed the DS’s internal consistency index of > 0.70 [3, 10, 11], which exceeds established threshold for reliability [12]. Additionally, an investigation of validity, reliability, and responsiveness of the DS in a large randomized placebo-controlled clinical trial demonstrated utility of the DS in this setting [13]. In assessing responsiveness (i.e., ability to detect change in patients receiving an intervention), change in total DS score between baseline and trial completion was found to be significantly correlated to change scores in other outcomes [13]. A study assessing criterion validity of the DS, comparing total DS score to another dependence-related measure (Official Scale for the Assessment of Dependence and Disability of the Spanish Ministry of Health, Social Policy and Equality (OSADD)) found the two dependence measures strongly correlated (*r* = 0.885; *p* < 0.001) [14].

**Table 3:** Validation studies assessing the psychometric properties of the DS

Study	Population	*n*	Outcome
Stern et al. 1994 [3]: Predictors I study	Mild–moderate AD	249	Inter-rater reliability (interclass correlation), 0.99 for total score; internal consistency (Cronbach’s α), 0.66; significant correlation with CDR (*r* = 0.34) and mMMS (*r* = − 0.27)
Lenderking et al. 2013 [10]: Predictors I + II studies	Mild-moderate AD	460	Internal consistency (Cronbach’s α), 0.72; construct validity, correlation with BDRS (r = 0.60), mMMS (*r* = − 0.30)
Lenderking et al. 2013 [10]; DADE Study	Mild-moderate AD	172	Internal consistency (Cronbach’s α), 0.72; construct validity, significant correlation with MMSE (*r* = − 0.47), CDR-SB (r = − 0.64), DAD (*r* = − 0.72), and NPI (*r* = 0.21)
Lenderking et al. 2013 [10]: ELN-AIP 901 study	Mild-moderate AD	166	Internal consistency (Cronbach’s α), 0.74; construct validity, significant correlation with MMSE (*r* = − 0.53), ADAS-cog (*r* = − 0.42), DAD (*r* = − 0.77), and NPI (*r* = 0.51)
Wyrwich et al. 2014 [13]	Mild-moderate AD	2334	Internal consistency (Cronbach’s α), 0.66; construct validity, significant correlation with ADAS-cog (*r* = 0.36), MMSE (r = − 0.34), DAD (*r* = − 0.63), CDR-SB (*r* = 0.61), and NPI (*r* = 0.32); responsiveness, significant correlation with change scores on ADAS-cog, DAD, CDR-SB, and MMSE (baseline to week 78)
Brickman et al. 2002 [18]: Predictors 1 study	Mild-moderate AD	230	Statistically significant change in total DS score over time (0.5 points per 6 month interval) (*p* < 0.001).
Garre-Olmo et al. 2015 [14]: CoDep AD study*	Mild-severe AD	343	Internal consistency (Cronbach’s α), 0.85; significant correlation with DAD score (*r* = 0.900), CBI (*r* = 0.323), NPI (*r* = 0.374), and MMSE (*r* = 0.617)

*CDR* clinical dementia rating, *MMSE* Mini Mental State Examination, *mMMS* modified MMSE, *BDRS* blessed dementia rating scale, *CDR-SB* Clinical Dementia Rating Scale Sum of Boxes, *DAD* disability assessment for dementia, *ADAS-cog* Alzheimer’s Disease Assessment Scale-cognitive subscale, *NPI* neuropsychiatric inventory, *CBI* caregiver burden interview. *Spanish translation of DS

### The Dependence Scale and other clinical measures of Alzheimer’s disease severity

#### Cognition, function, and behavior

Various studies have examined correlation between the DS and frequently used clinical measures of AD severity. Stern et al. [3] found that the dependence level at baseline significantly but weakly correlated with modified Mini Mental State Exam (mMMS; *r* = − 0.27), Clinical Dementia Rating (CDR) score (*r* = 0.34), and the cognitive (*r* = 0.38) and basic self-care factors (*r* = 0.26) of the Blessed Dementia Rating Scale (BDRS; *p* < 0.001). The cross-sectional DADE study in the UK showed a significant association between total DS score and cognition (MMSE; *r* = − 0.47), global disease severity (Clinical Dementia Rating Scale Sum of Boxes (CDR-SB); *r* = 0.64), function (Disability Assessment for Dementia (DAD); *r* = − 0.72), and a weaker association with behavior (Neuropsychiatric Inventory (NPI); *r* = 0.21) [11]. Moreover, this study showed that DAD, MMSE, and CDR-SB could explain 68%, 44%, and 59% of variation in total DS score, respectively (*p* < 0.01). In the combined Predictors studies at baseline, total DS score correlated strongest with BDRS (*r* = 0.60), while the Columbia University Scale of Psychopathology in Alzheimer’s disease (CUSPAD; *r* = 0.25) and mMMS (*r* = − 0.30) showed weaker correlations (all *p* < 0.0001) [10]. The ELN-AIP-901 study showed a similar correlation between total DS score and other measures at 26 and 78 weeks. The strongest correlation was with the DAD score (*r* = − 0.77 and *r* = − 0.74 at 26 and 78 weeks, respectively), followed by MMSE (*r* = − 0.53 and *r* = − 0.55 at 26 and 78 weeks, respectively) [10]. Similarly, after converting functional scores (ADCS-ADL) in a European cohort (GERAS study, *n* = 1497 AD patients) to dependence levels, it was found that higher levels of dependence correlated with decreased MMSE and NPI scores (*p* < 0.001) [15, 16]. The DS also was validated in a cohort of 343 Spanish participants, with a strong correlation between total DS score and DAD (*r* = − 0.90). It was also found that the relative contributions of cognitive, functional, and behavioral measures to total DS score change at various stages of the disease, supporting the unifying quality of dependence as a construct [14]. Similar to findings from the DADE study, DAD was the strongest predictor of total DS score variability (50.3% in mild AD and 64.2% in severe AD) in the Spanish-language version of the DS [14]. Similarly, a study using the Austrian PRODEM cohort of mild to moderate AD patients found the strongest correlation between total DS score and DAD (*r* = 0.79) followed by CDR (*r* = 0.54), NPI (*r* = 0.35), and MMSE (*r* = − 0.31) (all *p* < 0.001) [17].

Assessing the DS over time in a randomized placebo-controlled clinical trial setting, change in total DS score correlated to change scores in Alzheimer’s Disease Assessment Scale-cognitive subscale (ADAS-cog; *r* = 0.40), CDR-SB (*r* = 0.48), DAD (*r* = − 0.51), MMSE (*r* = − 0.45), and NPI (*r* = 0.41) [13].

Multiple studies have demonstrated the construct validity of the DS, showing significant increase in dependence over time, independent of global cognition and other self-care deficits, demonstrating the sensitivity of the DS to natural progression of AD [18, 19]. In the Predictors 1 study, an increase of 0.5 points per 6 months in the total DS score was observed [20]. Dependence levels increased by 0.26 after 6 months and 0.45 after 1 year [3]. In the combined Predictors 1 and Predictors 2 cohort a similar increase of 1.03 points in the total DS score per year was reported [21]. In another study of non-institutionalized AD patients, a worsening of 1.2 points was observed after 12 months of follow-up [22].

In the Predictors 1 study, a one-point change in the total DS score over 18 months was correlated with stability in living situation, i.e., remaining in own home or remaining in assisted living [10]. Patients that progressed in living situation, requiring higher levels of care (e.g., progressing from own home to assisted living) showed a larger increase in the total DS score, ranging from 2.31 to 5.81 points higher than those with a stable living situation. This was confirmed in a mild AD population (MMSE ≥ 21), in which an approximately one-point change in the total DS score was seen with stable living situation after 78 weeks; however, in the moderate AD (MMSE ≤ 20) subgroup of patients that remained at a stable living situation, an increase of 1.5–2 points in the DS score was observed.

### Dependence Scale and health economic outcomes

As the prevalence of the disease increases and cost of care continues to stress healthcare systems, cost considerations of current and novel therapies in AD are of high importance. Generally, cost of care of AD patients increases with greater disease severity. This holds true for severity captured by cognitive, functional, and behavioral measures [23].

A systematic review of methods of cost estimation in AD concluded that there was no established consensus on the appropriate method to model progression of AD for use in cost analyses [24]. A review of studies that examined the association between cognitive, functional, and behavioral endpoints and costs associated with AD found that cognition, function, and behavioral dysfunction each were independently associated with cost but any one of these measures alone did not fully represent healthcare cost [23]. Consequently, it is proposed that measures of cognition, function, and behavior be included in cost analyses simultaneously or, alternatively, a composite measure be used that captures deficiencies in each measure. Dependence on others as measured by the DS has been suggested as a unifying construct better suited than individual measures of cognition, function, and behavior to estimate the economic impact of AD [1, 19].

In the ELN-AIP-901 study cohort, the MMSE, DAD, and CDR were compared side-by-side to the DS to assess their utility in economic modeling [25]. Total DS score demonstrated the highest correlation to direct cost (R^2^ = 0.22, *p* = 0.004), followed by DAD (R^2^ = − 0.021, *p* = 0.006), while MMSE and CDR were not significantly correlated with cost. In addition, the DS and DAD had the highest correlation with caregiver time (R^2^ = − 0.52, *p* < 0.0001 and R^2^ = 0.49, *p* < 0.0001, respectively).

In several studies by Zhu et al. the association between dependence and cost was longitudinally examined in the Predictors study cohort [19, 26–28]. The DS was significantly associated with total cost. A one-point increase in the total DS score was associated with a US$1832 increase in total annual costs. The DS was found to have the strongest association with informal costs (caregiver time in assistance with activities of daily living (ADL)) [26]. In a separate study, dependence was significantly associated with medical costs (5.7% increase in cost per one DS point) and caregiver time (4.1% increase per one DS point) independent of functional impairment [24].

In cross-sectional analyses in European cohorts, the association between DS and cost was shown to be independent of function or other severity measures [29–31]. A one-point increase in the total DS score was correlated with a €796 increase in total cost in an Irish cohort of non-institutionalized patients with AD or MCI. In the same cohort followed for 2 years it was found that a one-point increase in the total DS score increased costs by 19%, mostly attributable to increases in informal care costs through caregiver time [29]. In the DADE study, a one-point increase in the total DS score was associated with £321 increase in cost of care, excluding unpaid care costs [11].

The Co-Dependence in Alzheimer’s Disease study in Spain assessed the association with multiple cost variables from dependence, time since diagnosis, comorbidities, and living situation. The DS was found to be the only measured variable independently associated with medical care cost, social care cost, indirect cost, and informal care cost [32]. A one-point increase in the total DS score was associated with a 13.5% increase in direct medical care costs, a 25.3% increase in social care costs, and a 214.7% increase in informal care costs of 6 months.

Using data from the Predictors 1 study, it was demonstrated that the DS was useful in prospectively predicting home health aide (HHA) use [33]. The annual probability of transitioning to needing a HHA increased as the total DS score increased, from 4% at a DS score of 1 to 54% at a DS score of 10. Specifically, three items of the DS (needing household chores done for oneself, needing to be watched or kept company when awake, and needing to be escorted when outside) were found to be significant predictors of the presence of an HHA.

#### Patient quality of life

The ability of an AD severity measure to bridge AD symptomatology and quality of life (QoL) outcomes is of particular importance when determining its utility in assessing disease progression and guiding policy [25]. In the ELN-AIP-901, total DS score showed the highest correlation with Quality of Life-Alzheimer’s Disease (QoL-AD; *r* = − 0.52, *p* < 0.0001) compared to MMSE, DAD, and CDR scores (*r* = 0.25, *r* = 0.48, and *r* = 0.39, respectively; all *p* < 0.0001). Total DS score explained the greatest variance in QoL (R^2^ = 0.33) compared to DAD, CDR, and MMSE [25]. Several other cross-sectional analyses have shown decreased QoL with increasing dependence [15]. In the cross-sectional DADE study, QoL measured by both self-report and proxy scales generally were negatively associated with the DS [11]. The longitudinal change in QoL assessed in DADE-2, however, was not associated with DS or any other measure of severity [34]. This is potentially attributable to the large variation in QoL in this study and a significant percentage of participants that reported a paradoxical improvement in QoL after 18 months.

### Dependence Scale as a unifying measure

While the DS is strongly associated with other clinical endpoints in AD, variance in the total DS score is incompletely explained by cognitive, functional, and behavioral impairment alone [11, 17]. In an assessment of the DS in the Predictors study cohort, the DADE cohort and the ELN-AIP-901 cohort, only 50% of variance in the total DS score was explained by cognitive, functional, and behavioral scales [10]. Others show that functional and behavioral measures combined with age and other comorbidities fail to predict 26.0–40.2% of variability in the total DS score, depending on AD severity [14]. The Predictors study supported the distinctness of the dependence construct from other AD severity measures, finding that total DS score changes over time after correcting for changes in cognitive and functional scores [18, 33].

Several studies used path models to examine the complex relationships between key measures of AD, including behavior, cognition, functioning, and dependence, and examined distinctiveness of each measure and strengths of these relationships [13, 31, 35]. Wyrwich and colleagues examined reliability, validity, and responsiveness of the DS in a large randomized controlled trial of patients with mild to moderate AD and showed that not all of the variation in total DS score was explained by these other key measures of AD. Two studies further examined the relationships between different factors affecting costs in dementia patients [31, 35]. In both studies, dependence, measured by the DS, was modeled as an endogenous variable, mediating the effect of various clinical characteristics, including cognitive, function, behavioral measures, and comorbid conditions. Results showed that measures of cognition, behavior, and other factors significantly predicted total DS score, which in turn predicted total AD-related costs. Despite differences in the study sample and measures used, both studies showed that the significant impact that these factors have on costs were largely mediated by dependence. Together, these models support dependence as a distinct, measurable component of dementing disease, which ought to be an important outcome in studies of AD.

## Conclusions

The DS provides a measurement of AD patients’ level of dependence and assesses aspects of life critical to patient autonomy. This includes the assessment of what needs to be done for the patient, which is related to limitations in cognition and function, but captures core aspects of patients’ experience with AD that are distinct from and more comprehensive than these individual measures of disease progression.

The psychometric properties of the DS have been well established in over two decades since its creation. The DS has been broadly assessed in patients with AD and provides meaningful and unique information on patient needs and independence, rather than on the ability to perform specific tasks. By going beyond individual components of disease severity, the DS has specific utility in complementing scales that assess cognition, function, and behavior. Longitudinal studies have shown progression in dependence over time, and the DS has also been shown to correlate with numerous other scales that assess distinct concepts of cognition, function, and behavior in AD. While the DS and other clinical endpoints in AD are strongly correlated, clinical scales that capture these endpoints alone incompletely drive change in the DS. Thus, variance in the DS is poorly explained by cognitive, functional, and behavioral impairment alone. In addition, the DS has shown meaningful associations with other metrics relevant to stakeholders in AD, including QoL metrics and caregiver burden. The DS has also proven to be strongly correlated to various cost aspects of AD, suggesting it is a valuable unifying construct in determining value to patients in economic assessments of interventions.

Clinical trials in AD need validated and reliable measures of disease severity that capture disease impact at early (cognitive) and late (functional and/or behavioral) stages, are sensitive to change over time, and are transparent to all stakeholders. Most commonly used AD scales predominantly measure severity in individual domains of dysfunction. They are not sufficiently sensitive to all AD stages, and are therefore not easily applicable for tracking changes in routine medical practice or comparable in different patient populations. To date, a number of randomized controlled trials have used the DS to characterize baseline need for care and to assess the efficacy of therapeutic interventions in AD. Specifically, the DS has been incorporated as a secondary endpoint in AD trials exploring the efficacy of bapineuzumab [36], selegiline [37], α-tocopherol [38], memantine [38], antipsychotics [39], and statins [40] in patients with varying severities of AD.

Overall, findings presented in this review support the notion that dependence, reflected in the psychometrically robust DS, is related to, but distinct from, key severity measures, including cognition, function, and behavior, captures the full spectrum of patient needs, and is independent of sociocultural influences. Dependence also presents as a useful measure for assessing disease progression as it relates to cost of care, operating through unifying multiple severity measures into a single, comprehensive scale. This underscores the importance of the DS as a unique endpoint in AD clinical trials, providing important information about the impact of therapeutic interventions on a measure of relevance to patients, caregivers, and other stakeholders involved in the care of patients with AD. Interventions that reduce AD patients’ dependence are expected to contribute to reduction in costs of care. The DS is therefore a useful measure for economic evaluation of novel interventions aimed at delaying progression to higher levels of dependence.

## Abbreviations

AD: Alzheimer’s disease
ADAS-cog: Alzheimer’s Disease Assessment Scale–cognitive subscale
BDRS: Blessed Dementia Rating Scale
BGP: Behavioral Rating Scale for Geriatric Patients
CDR: Clinical Dementia Rating
CDR-SB: Clinical Dementia Rating Scale Sum of Boxes
CUSPAD: Columbia University Scale of Psychopathology in Alzheimer’s disease
DAD: Disability Assessment for Dementia
DS: Dependence Scale
ILS: Independent Living Scales
MMSE: Mini Mental State Exam
NCD: Nursing Care Dependency
NPI: Neuropsychiatric Inventory
OSADD: Official Scale for the Assessment of Dependence and Disability of the Spanish Ministry of Health Social Policy and Equality
QoL-AD: Quality of Life-Alzheimer’s Disease
RIL: Record of Independent Living
